# RETRACTED: Patel et al. Effect Comparison of E-Cigarette and Traditional Smoking and Association with Stroke—A Cross-Sectional Study of NHANES. *Neurol. Int.* 2022, *14*, 441–452

**DOI:** 10.3390/neurolint17120207

**Published:** 2025-12-18

**Authors:** Urvish Patel, Neel Patel, Mahika Khurana, Akshada Parulekar, Amrapali Patel, Juan Fernando Ortiz, Rutul Patel, Eseosa Urhoghide, Anuja Mistry, Arpita Bhriguvanshi, Mohammed Abdulqader, Neev Mehta, Kogulavadanan Arumaithurai, Shamik Shah

**Affiliations:** 1 Department of Public Health, Icahn School of Medicine at Mount Sinai, New York, NY 10029, USA; neel4ahm@gmail.com; 2 Department of Public Health, University of California, Berkeley, CA 92093, USA; mahika_khurana@berkeley.edu; 3 Department of Biology, York University, Toronto, ON M3J 1P3, Canada; akshadapar@gmail.com; 4 Department of Public Health, Milken Institute School of Public Health, George Washington University, Washington, DC 20052, USA; patelamrapali428@gmail.com; 5 Department of Neurology, Larkin Community Hospital, Miami, FL 33143, USA; sumjuanfer41@gmail.com; 6 Department of Internal Medicine, Texas Tech University Health Sciences Center, Transmountain Campus, El Paso, TX 79430, USA; rutul.patel@ttuhsc.edu (R.P.); anumistr@ttuhsc.edu (A.M.); 7 Department of Internal Medicine, Hudson Regional Hosp, Secaucus, NJ 07094, USA; eseosaurhoghide@gmail.com; 8 Department of Child Neurology, Jersey Shore University Medical Center, Neptune City, NJ 07753, USA; bhriguva@gmail.com; 9 Department of Internal Medicine, Geisinger Med Center, Danville, PA 17822, USA; mhdaliabdulqader@gmail.com; 10 Department of Epidemiology and Biostatistics, Boston University School of Public Health, Boston, MA 02118, USA; neev.n.mehta@gmail.com; 11 Department of Neurology, Mayo Clinic, Albert Lea, MN 56007, USA; dockogul26@gmail.com; 12 Department of Neurology, Stormont Vail Health, Topeka, KS 66604, USA; drshahshamik@gmail.com

The journal retracts the article, “Effect Comparison of E-Cigarette and Traditional Smoking and Association with Stroke—A Cross-Sectional Study of NHANES” [1], cited above.

Following publication, concerns were brought to the attention of the publisher regarding several major errors in the data analysis, raising concerns about the validity of the findings.

Adhering to our complaint procedure, an investigation was conducted by the Editorial Office and Editorial Board. Although the authors initially cooperated, they were unable to provide satisfactory explanations or supporting material to resolve the issues identified. Furthermore, attempts to contact the relevant institution for further information were unsuccessful, as no response was received. As a result, the Editorial Board and Editor-in-Chief have lost confidence in the reliability of the findings and have decided to retract the article [1] as per MDPI’s retraction policy (https://www.mdpi.com/ethics#_bookmark30).

This retraction was approved by the Editor-in-Chief of the journal *Neurology International*.

The authors did not agree to this retraction.
